# 

**Published:** 1887-05-21

**Authors:** 


PRICE ONE PENNY.

No. 34, Vol. II.

May 21st, 1887.

[Registered at the General Post Office as a Newspaper.]

Per Annum, 6s. 6d., post free.

Monthly Parts, 6d.; post free, 7½d.

Ditto per Ann., 7s. 6d., post free.

